# Image-Based Recognition of Intricate Animal Motifs on Ming Dynasty Blue and White Porcelain Using an Improved YOLOv8n Model

**DOI:** 10.3390/s26144457

**Published:** 2026-07-14

**Authors:** Yaqing Zhao, Shunren Luo, Qiang Wang, Qihao Sun

**Affiliations:** 1 School of Design, Jiangnan University, Wuxi 214000, China; 7230306017@stu.jiangnan.edu.cn; 2 School of Art and Design, Nanjing Institute of Technology, Nanjing 211167, China; j00000003741@njit.edu.cn; 3 School of Information, Renmin University of China, Beijing 100872, China

**Keywords:** YOLOv8n, deformable convolution networks v2, efficient multi-scale attention, MPDIoU loss function, image-based recognition, animal motif recognition, Ming Dynasty blue and white porcelain

## Abstract

**Highlights:**

- YOLOv8n is applied to the recognition of intricate decorative motifs, expanding its application from general object detection to ceramic image analysis.
- A dedicated annotated image dataset, targeted data augmentation strategies, and three model optimization mechanisms are combined to improve the recognition accuracy of animal motifs on Ming Dynasty Blue and White Porcelain.

**What are the main findings?**

- The improved YOLOv8n model achieved 96.4% mAP@0.5 and 80.8% mAP@0.5:0.95, outperforming the baseline YOLOv8n model.
- The integration of DCNv2, EMA, and MPDIoU enhanced the model’s ability to detect deformed motifs and suppress interference from complex decorative backgrounds.

**What are the implications of the main findings?**

- The proposed method provides a non-destructive and real-time approach for recognizing intricate decorative motifs in cultural heritage images.
- The model shows potential for future deployment on mobile or edge devices to support digital preservation, museum collection management, archaeological analysis, and image-based visual sensing applications.

**Abstract:**

Image-based recognition of intricate decorative motifs in complex visual environments remains a challenging task in computer vision due to geometric deformation, scale variation, illumination changes, and background interference. These challenges are particularly evident in images of animal motifs on Ming Dynasty Blue and White Porcelain, where curved vessel surfaces and decorative complexity significantly increase recognition difficulty. To facilitate robust model training and evaluation, this study establishes a dedicated annotated image dataset of animal motifs and expands it through targeted data augmentation strategies. Furthermore, an improved YOLOv8n-based object detection framework is proposed, featuring three key optimizations: (1) the CBS modules in the backbone and neck networks are replaced with Deformable Convolution Networks v2 (DCNv2) to strengthen the model’s feature extraction capability for deformed motifs; (2) an Efficient Multi-Scale Attention (EMA) mechanism is introduced into the neck network to integrate multi-scale features and suppress interference from complex decorative backgrounds; and (3) the original CIoU loss function is replaced with the MPDIoU loss function to improve localization accuracy and convergence speed. Experimental results demonstrate that the improved model achieves mAP@0.5 and mAP@0.5:0.95 values of 96.4% and 80.8%, respectively, representing improvements of 1.7% and 3.2% over the baseline model, while maintaining a detection speed of 89.3 FPS. These results indicate that the proposed framework provides an accurate, non-destructive, and efficient image-based method for cultural heritage object recognition. It can support museum collection management, archaeological analysis, and image-based visual sensing applications, while also showing potential for further optimization and deployment on compact edge-computing platforms.

## 1. Introduction

Blue and White Porcelain of the Ming Dynasty represents an important stage in the history of Chinese ceramics. As a type of porcelain decorated with underglaze cobalt blue, its mature firing techniques and highly recognizable visual style have established it as one of the most representative ceramic forms in Chinese material culture [1]. Blue and White Porcelain uses cobalt oxide as its coloring agent; after patterns are painted on the porcelain body, a transparent glaze is applied, and the piece is fired at high temperatures in a single firing, producing a distinctive visual contrast between cobalt blue and white [2]. These technical characteristics not only established its significant status in the history of ceramic technology but also enabled it to serve as an important material medium for transnational trade, supporting cultural exchange between China and other regions [3].

**Figure 1:**
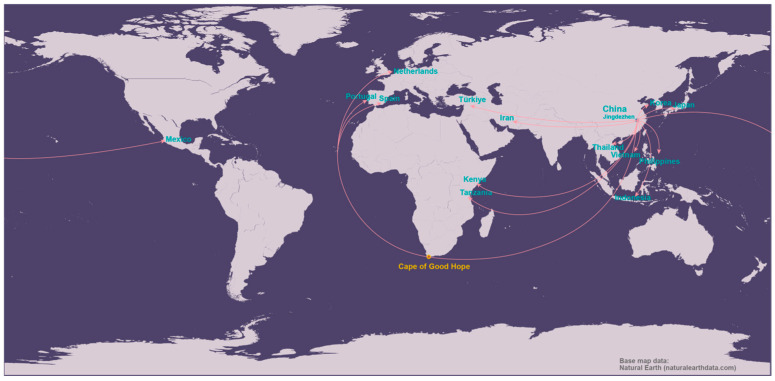
Transnational trade routes of Ming Dynasty Blue and White Porcelain.

Jingdezhen Blue and White Porcelain from the Ming Dynasty features a rich variety of decorative motifs and styles. Through long-term transmission and dissemination, a substantial body of visual resources has been accumulated, providing a foundation for digital documentation, ceramic image analysis, and automatic motif recognition. Within its decorative system, motifs exhibit a high degree of diversity, with animal motifs being particularly representative. These include a wide range of types, such as dragons, phoenixes, fish, cranes, and lions, and constitute a significant component of the visual imagery of Blue and White Porcelain. Furthermore, through long-distance trade and cultural exchange, these motifs exerted a lasting influence on the imitation, adaptation, and localized transformation of related decorative traditions in countries and regions such as Japan, Korea, Vietnam, Iran, Türkiye, the Netherlands, Egypt, and Mexico, where they became important sources of artistic inspiration for local ceramic and decorative arts [4,5,6,7] (Figure 1).

Research on animal motifs has mainly been conducted within the frameworks of traditional ceramic history and art history, with methodologies dominated by typological analysis, archaeological dating, and stylistic interpretation. Related studies largely focus on the periodization of vessels, the evolution of decorative themes, and visual stylistic characteristics. By integrating excavated materials with handed-down artifacts, researchers have discussed the cultural symbolic meanings and technical characteristics of these motifs [8,9,10,11]. Overall, existing research on Blue and White Porcelain motif imagery relies heavily on manual empirical judgment and static image comparison, focusing predominantly on the elucidation of historical contexts and aesthetic features. Although substantial progress has been made in artifact categorization and image description, the overall research paradigm remains qualitative and has yet to establish a data-driven analytical framework for large-scale image processing and automatic motif recognition.

With the continuous development of digital technologies, non-destructive analytical techniques have provided precise methods for the scientific identification of ancient ceramics and have improved detection efficiency. In the field of spectral analysis, various spectroscopic techniques, including X-ray fluorescence (XRF) spectroscopy, Raman spectroscopy, and laser-induced breakdown spectroscopy (LIBS), have been employed to detect the chemical composition of ancient ceramics [12,13,14,15,16]. Concurrently, the integration of spectral techniques with machine learning algorithms, such as Random Forest, Support Vector Machines (SVM), and k-Nearest Neighbors (KNN), has facilitated the efficient classification and provenance tracing of these artifacts [17,18]. Furthermore, generative artificial intelligence has been applied to the digital reconstruction and restoration of ancient ceramics, providing an additional technical approach for addressing the inefficiencies of traditional manual restoration and supporting the digital preservation of cultural heritage [19,20,21].

For image-based recognition tasks, deep learning architectures represented by Convolutional Neural Networks (CNNs) have reduced the dependence on manual feature extraction and have become a mainstream paradigm in computer vision [22]. Researchers have employed models such as VGG, ResNet, Inception, ShuffleNet, and YOLO for vessel-shape classification, image recognition, glaze-color recognition, and dating of ancient ceramics [23,24,25,26]. Among them, the YOLO (You Only Look Once) series, as a typical single-stage object detection algorithm, has achieved an effective balance between inference speed and detection accuracy [27]. This algorithm transforms object detection into a bounding box regression problem and a class probability classification problem, achieving end-to-end training and detection, thereby enhancing detection accuracy and robustness [28]. In particular, YOLOv8, with its anchor-free mechanism and decoupled head structure, reduces mutual interference between tasks, significantly enhancing the model’s overall performance and lightweight deployment capability [29]. Consequently, YOLOv8 has been widely validated in various practical application scenarios, such as vehicle detection, pedestrian detection, smart orchards, animal husbandry, ship tracking, and material defect detection [30,31,32,33,34,35]. In recent years, the YOLOv8 model has been introduced into the field of cultural heritage, with research primarily unfolding in two aspects. The first focuses on the intelligent detection and sustainable protection of architectural heritage, such as roof damage detection in ancient villages, wood structure damage detection in Fujian Tulou, crack detection in fair-faced brick walls, and fire detection in ancient buildings [36,37,38,39]. The second focuses on the image recognition of various cultural heritage carriers, covering ceramics, murals, statues, embroidery images, and Yunnan Jiama [40,41,42,43,44]. These studies indicate that YOLOv8 can provide efficient and relatively low-cost technical support for cultural heritage image analysis, while also demonstrating the generalization capability of the YOLOv8 architecture in recognizing domain-specific visual objects.

However, existing research on ancient ceramic image recognition based on YOLOv8 remains at an early stage. It is still mainly limited to coarse-level recognition or classification of macroscopic vessel shapes [40], and has not yet sufficiently addressed detection tasks targeting intricate decorative motifs on Blue and White Porcelain. In particular, animal motifs are frequently situated against complex monochrome backgrounds. Their morphologies are highly diverse, and their edges are often indistinct due to the diffusion and blurring of the underglaze color. These characteristics impose stringent requirements on the model’s capability for local detail feature modeling, multi-scale feature fusion, and accurate localization. Accordingly, this study proposes an improved YOLOv8n-based object detection framework for recognizing and localizing animal motif categories in Ming Dynasty Blue and White Porcelain images. This study aims to address the lack of object detection research on intricate motifs in ceramic images and to provide a technical pathway for structured analysis and retrieval of ceramic image data.

The main contributions of this study are as follows:

- A dedicated annotated image dataset of animal motifs on Ming Dynasty Blue and White Porcelain is constructed and expanded through targeted data augmentation strategies. The dataset covers representative motif categories, vessel forms, preservation conditions, and imaging perspectives, providing a data basis for image-based recognition of intricate animal motifs in ceramic images.
- An improved YOLOv8n-based object detection framework is developed for the recognition and localization of animal motifs. By incorporating DCNv2, EMA, and MPDIoU, the model enhances feature extraction for deformed motifs, strengthens multi-scale feature fusion, suppresses interference from complex decorative backgrounds, and improves localization accuracy.
- The proposed model is systematically evaluated using quantitative metrics and ablation studies. The results demonstrate the effectiveness of the improved model for image-based recognition of intricate animal motifs under complex visual conditions, including geometric deformation, scale variation, illumination changes, and background interference.

## 2. Materials and Methods

### 2.1. Sample Collection and Dataset Construction

**Figure 2:**
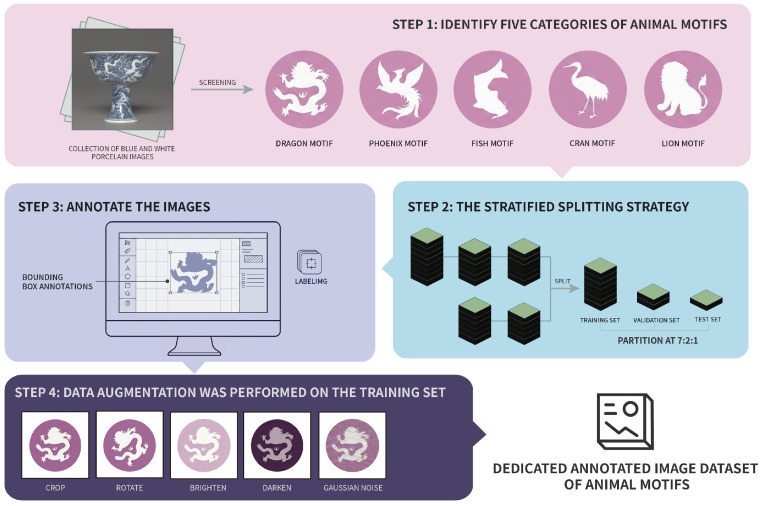
Construction workflow of the dedicated annotated image dataset of animal motifs.

This section describes the construction process of a dedicated annotated image dataset of animal motifs. First, raw images collected from multiple sources were screened to identify five representative categories of animal motifs. Second, the selected original images were divided into training, validation, and test sets according to a stratified splitting strategy, and the distribution of original images across the three subsets was recorded. Third, precise bounding box annotations were performed using LabelImg, version 1.8.6, and were further reviewed to improve annotation accuracy and consistency. Finally, targeted data augmentation strategies were applied only to the training set to increase image diversity. Through this process of systematic screening, stratified partitioning, annotation, and training-set augmentation, a dedicated annotated image dataset of animal motifs was established, providing a reliable data basis for subsequent deep learning model training and performance evaluation (Figure 2).

#### 2.1.1. Construction of the Initial Dataset

This study constructed a dedicated annotated image dataset for the recognition of animal motifs on Ming Dynasty Blue and White Porcelain. All images were manually collected and carefully screened. The image sources included representative museum digital collections, such as those of the National Palace Museum in Taipei, The British Museum, and The Metropolitan Museum of Art, as well as professional catalogs published by authoritative institutions and firsthand images acquired from relevant exhibitions. After systematic screening, dragon, phoenix, fish, crane, and lion motifs were selected as the five target categories because they are well represented in surviving examples, display rich stylistic variation, and are widely used across different vessel forms. These motif categories also carry cultural symbolic meanings and ritual associations. The initial image dataset contained 831 original images, including 335 dragon motif images, 146 phoenix motif images, 128 fish motif images, 116 crane motif images, and 106 lion motif images, covering different motif forms, vessel types, and preservation conditions (Table 1). During dataset splitting, the images in each animal motif category were independently divided into training, validation, and test sets at a ratio of 7:2:1, and the corresponding subsets were then aggregated. As shown in Table 1, the 831 original images were divided into 580 training images, 166 validation images, and 85 test images. This stratified splitting strategy helped maintain a relatively consistent class distribution across the three subsets. To reduce the potential risk of data leakage, image sources, vessel information, and motif similarity were further checked manually, so as to minimize the possibility that images from the same vessel or the same identifiable motif source were assigned to different data subsets.

The subsequent data annotation stage was then conducted using LabelImg. Bounding boxes were delineated around the animal motifs appearing in each image, and each bounding box was assigned the corresponding category label. To improve annotation accuracy and consistency, the annotations were first completed by two doctoral students specializing in ceramic history and one researcher specializing in computer vision. The annotations were then reviewed by three ceramic research specialists, with attention to category identification, bounding-box boundaries, and ambiguous images. Through this process, the standardization and reliability of the image annotations were improved, providing a data basis for subsequent model training and performance evaluation.

#### 2.1.2. Data Processing Strategies

Data augmentation is an important strategy for improving the generalization capability and robustness of object detection models. By expanding the training samples and introducing controlled perturbations, it enables the model to learn richer visual features. To improve the adaptability of the model to animal motifs on Ming Dynasty Blue and White Porcelain under multi-scale, multi-view, and complex visual conditions, this study applied targeted data augmentation strategies to the training set, expanding the training samples to 1532 images. Specifically, four augmentation methods were adopted: random cropping, random rotation, brightness adjustment, and Gaussian noise. The specific functions are detailed as follows:

- Random cropping was used to simulate field-of-view restrictions caused by changes in shooting distance or partial occlusion, thereby improving the model’s ability to capture multi-scale features.
- Random rotation was used to reduce the model’s dependence on fixed motif orientations and to address pose variations caused by curved vessel surfaces and multi-angle image acquisition.
- Brightness adjustment was used to linearly change pixel intensity to simulate different illumination conditions, including museum spotlights, natural light, and low-illumination environments.
- Gaussian noise was introduced to generate random fluctuations in pixel values and simulate image sensor noise and acquisition perturbations.

Together, these targeted data augmentation strategies improved the diversity of the training samples and helped the model achieve more reliable recognition performance under typical challenges, including complex backgrounds, partial defects, and illumination variations (Table 2).

### 2.2. Improvements to the YOLOv8n Model

With the continuous evolution of the YOLO series, YOLOv8 has achieved further optimizations in network architecture and feature extraction capability. It inherits the advantages of high efficiency and detection accuracy from earlier YOLO models and maintains stable performance in object detection tasks. To address different application requirements, the YOLOv8 series includes five versions: YOLOv8n, YOLOv8s, YOLOv8m, YOLOv8l, and YOLOv8x. Among these, YOLOv8n is a compact model with relatively low computational cost and fast inference speed, making it potentially suitable for mobile deployment and high-frame-rate scenarios [45]. The overall network architecture of YOLOv8n consists of three main components: the Backbone, the Neck, and the Head. The Backbone extracts foundational features from the input images. By replacing the C3 module in YOLOv5 with the C2f module, it introduces additional parallel branches and feature reuse mechanisms, thereby improving feature learning capability without a substantial increase in computational cost. The Neck fuses features from different hierarchical levels of the Backbone. By introducing the C2f module and reducing redundant convolutions, it decreases computational overhead while maintaining multi-scale feature fusion capability. The Head adopts a decoupled structure, processing classification and bounding box regression through two separate branches to reduce task interference.

**Figure 3:**
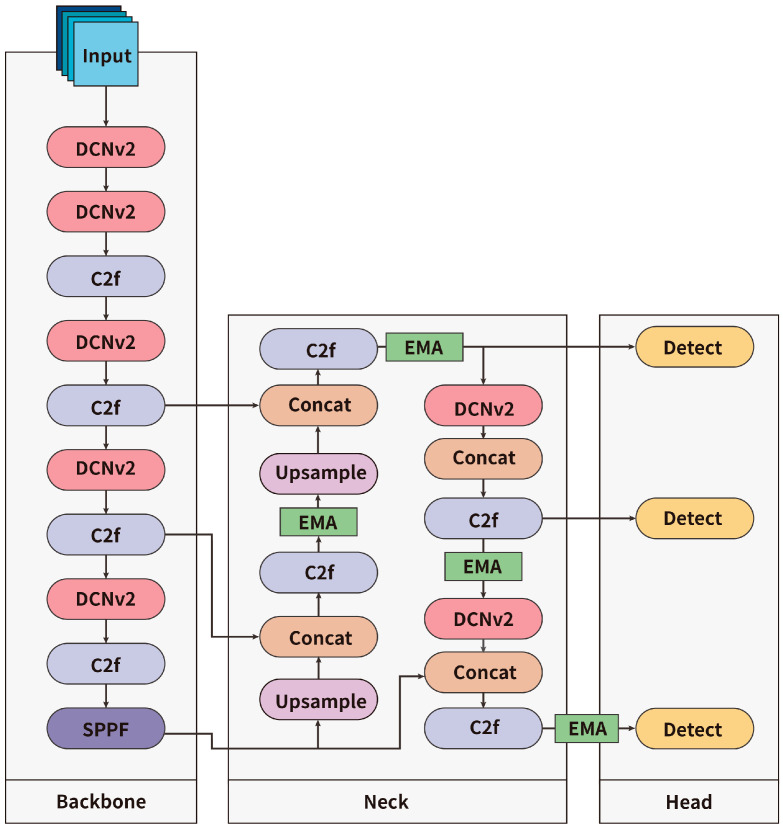
Architecture of the improved YOLOv8n model.

However, animal motifs on Ming Dynasty Blue and White Porcelain often exhibit diverse and irregular painted forms and are frequently embedded in complex decorative backgrounds. Therefore, the original YOLOv8n model may not fully capture the local details, geometric deformation, and multi-scale characteristics of these intricate motifs. To address these challenges, this study proposes targeted improvements to YOLOv8n in three aspects. First, selected CBS modules in the Backbone and Neck are replaced with Deformable Convolution Networks v2 (DCNv2) to enhance geometric modeling capability for irregular motif deformation while introducing limited computational overhead. Second, the Efficient Multi-Scale Attention (EMA) mechanism is embedded into the Neck to strengthen the model’s ability to capture motif information across different scales and suppress background interference. Third, the CIoU loss function is replaced with the MPDIoU loss function to improve localization accuracy and accelerate model convergence. The architecture of the improved model is illustrated in Figure 3.

This study improves the original YOLOv8n model from the three aforementioned aspects. The specific implementation schemes are detailed as follows.

#### 2.2.1. Deformable Convolution Networks v2 (DCNv2)

**Figure 4:**
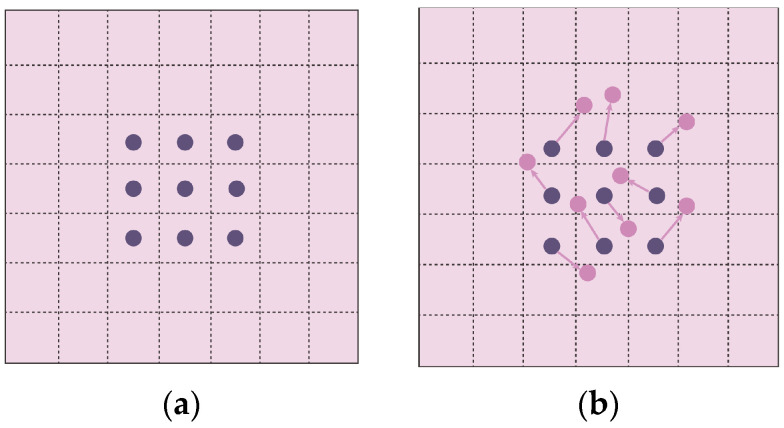
Comparison of sampling locations: (**a**) standard regular grid; (**b**) offset-adjusted locations in deformable convolution. Dark dots indicate the original regular sampling locations, while light purple dots indicate the offset-adjusted sampling locations.

**Figure 5:**
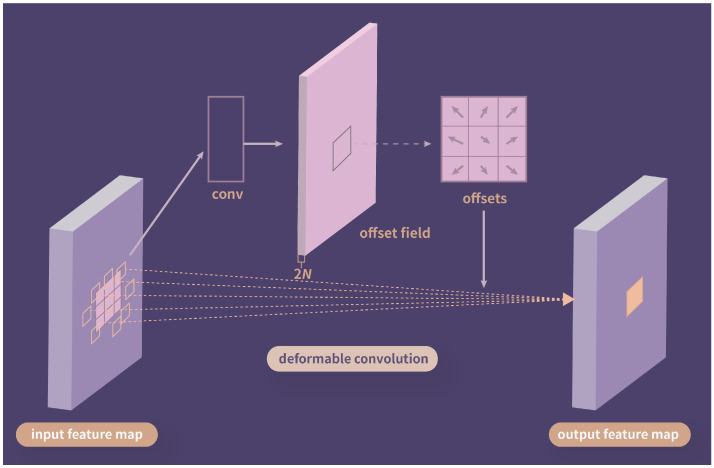
Structural diagram of the deformable convolution operation.

Standard convolution modules use rectangular sampling grids with fixed sizes and shapes, which limits their ability to process targets with complex geometric deformation. To address this issue, Deformable Convolution Networks (DCN) introduce learnable offsets to enhance the model’s geometric modeling capability. This mechanism allows the receptive field to adaptively adjust its shape and size according to the actual geometric structure of the image, thereby better fitting target motifs with irregular contours (Figure 4). Specifically, given an input feature map, the network first applies a standard convolutional layer to generate a spatial offset field. If a standard convolution kernel contains N sampling locations, the corresponding offset field has 2N output channels, which encode the offsets of each sampling location along the x- and y-axes. During deformable convolution, these offsets are added to the original regular sampling grid, producing an irregular distribution of actual sampling locations. This process allows the effective receptive field of the convolution kernel to adapt to the geometric shapes and scale variations of target objects. Such a dynamic sampling mechanism improves feature extraction accuracy and robustness in complex visual recognition tasks [46] (Figure 5).

DCNv2 represents a further optimized convolution operation. Building upon DCNv1, it introduces an additional learnable modulation scalar, $\Delta m_{n}$, for each sampling location. This allows the network to control the response intensity of individual sampling points and suppress irrelevant background information. In addition, by increasing the number of deformable convolutional layers, the model’s geometric modeling capability for complex deformations is further enhanced. This dual mechanism enables the model to dynamically weight feature responses according to the semantic importance of target regions while adaptively adjusting sampling locations. As a result, irrelevant background information can be filtered out more effectively, improving the discrimination and extraction of motif features in complex decorative backgrounds [47]. The output feature value, $y\left(p_{0}\right)$, is calculated using the following formula:

(1) $$y\left(p_{0}\right)=\sum_{p_{n}\in R}w\left(p_{n}\right)\cdot x\left(p_{0}+p_{n}+\Delta p_{n}\right)\cdot \Delta m_{n}$$

where $w\left(p_{n}\right)$ denotes the weight of the convolution kernel at location $p_{n}$, x represents the input feature map, $\Delta p_{n}$ denotes the offset superimposed on the regular sampling location, and $\Delta m_{n}$ is the corresponding modulation scalar for that sampling location. Given that the coordinates of the sampling locations after the superposition of offsets are typically non-integers, their actual pixel values are computed employing a bilinear interpolation algorithm.

#### 2.2.2. Efficient Multi-Scale Attention (EMA)

Attention mechanisms in YOLOv8 have been shown to improve the detection of fine visual details under challenging visual conditions, including small or inconspicuous targets, richly textured surfaces, complex backgrounds, and foreground–background interference. By enhancing sensitivity to target regions and reducing feature confusion, these mechanisms can improve both classification and localization performance [48]. Among these attention-based approaches, the Efficient Multi-scale Attention (EMA) mechanism is a lightweight improvement based on Coordinate Attention (CA). Its core objective is to achieve the efficient modelling of cross-spatial dimensions and multi-scale feature information without undertaking channel dimensionality reduction. Through a multi-scale parallel sub-network structure and a cross-spatial information aggregation strategy, EMA enables the model to further enhance local detailed features whilst preserving the global context. This facilitates the deep fusion of the global context and local motif information, thereby elevating the object detection capability in complex visual scenarios [49].

**Figure 6:**
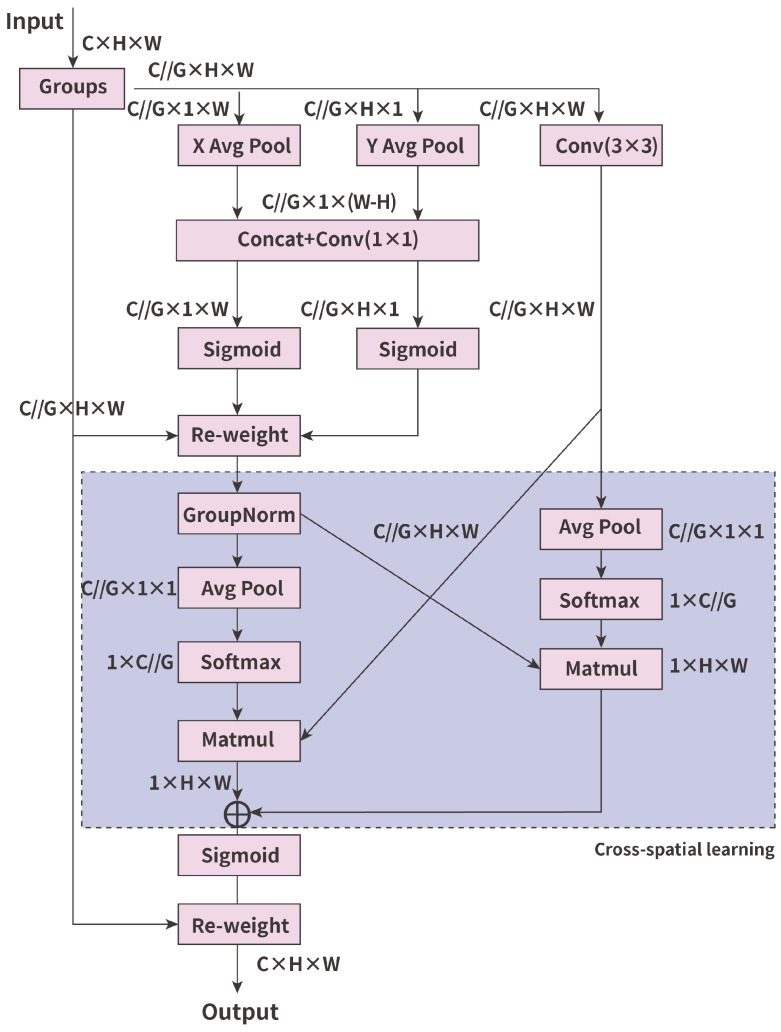
Structural diagram of the Efficient Multi-scale Attention (EMA) mechanism.

Specifically, EMA initially divides the input feature map $X\in R^{C\times H\times W}$ along the channel dimension into G mutually non-overlapping sub-feature groups $X_{i}\in R^{\left(C//G\right)\times H\times W}$. It then maps them to the batch dimension via dimensional restructuring to learn diversified semantic features across different sub-spaces. During the feature encoding phase, EMA constructs a parallel structure comprising a 1×1 branch and a 3×3 branch. The 1×1 branch employs two 1D global average pooling operations to aggregate features along the horizontal and vertical directions, respectively. This models long-range dependencies across different spatial directions whilst retaining precise positional encoding information. Subsequently, the encoded features from both directions are fused through a shared 1×1 convolution, and a Sigmoid function is utilized to generate the corresponding channel attention weights. Simultaneously, 3×3 the convolutional branch focuses on local spatial feature extraction, aiming to compensate for the deficiencies of global encoding in perceiving details such as motif edges and local brushstrokes. Building upon this, EMA further introduces a ‘cross-spatial learning’ strategy. It performs global spatial information encoding for the outputs of the different branches via 2D global average pooling, followed by dynamic weighting in conjunction with Softmax normalization. Finally, through the cross-weighted summation of the outputs from both branches, the spatial information from different parallel pathways is aggregated. The ultimate attention map is then generated via a Sigmoid activation function. This mechanism achieves the adaptive enhancement of crucial target regions and important motif features (Figure 6).

#### 2.2.3. MPDIoU Loss Function

In the bounding box regression task, YOLOv8n by default employs CIoU as the loss function to evaluate the similarity between the predicted box and the ground truth box. This function calculates the loss by comprehensively considering the overlapping area, the distance between the center points, and the aspect ratio of the predicted and ground truth boxes. Its calculation formula is as follows:

(2) $$L_{CIoU}=1-IoU+\frac{\rho^{2}\left(b_{gt},b_{pred}\right)}{C^{2}}+\alpha v$$

(3) $$IoU=\frac{B_{gt}\cap B_{pred}}{B_{gt}\cup B_{pred}}$$

(4) $$v=\frac{4}{\pi^{2}}{\left(\arctan\;\frac{w_{gt}}{h_{gt}}-\arctan\;\frac{w_{pred}}{h_{pred}}\right)}^{2}$$

(5) $$\alpha =\frac{v}{\left(1-IoU\right)+v}$$

where $B_{gt}$ denotes the ground truth box and $B_{pred}$ represents the predicted box; $b_{gt}$ and $b_{pred}$ are the center points of the ground truth box and the predicted box, respectively; $\rho^{2}\left(b_{gt},b_{pred}\right)$ represents the squared Euclidean distance between the center points of the predicted and ground truth boxes; C denotes the diagonal length of the smallest enclosing bounding box covering both the predicted and ground truth boxes; v is a parameter measuring the consistency of the bounding box aspect ratio; and α is a weight coefficient balancing the overlapping area and the aspect ratio penalty term.

Although the CIoU loss function handles the scale variations of bounding boxes relatively well, its aspect ratio penalty term focuses solely on the relative consistency in proportion between the predicted box and the ground truth box. When the aspect ratios of the predicted and ground truth boxes are identical, regardless of the differences in their actual dimensions, v becomes 0. Consequently, the penalty term fails, and the optimization process degrades to the DIoU loss, which only considers the distance between the center points. MPDIoU adopts the coordinates of the top-left and bottom-right corners of the bounding box as the core measurement standard. By directly minimizing the distance between the corresponding corner points of the predicted and ground truth boxes to construct constraints, it effectively improves the alignment accuracy of the bounding boxes [50]. In terms of geometric measurement, MPDIoU alleviates the degradation problem that occurs when bounding boxes have identical aspect ratios. Through corner distance constraints, the model can more directly perceive the scale and positional differences between bounding boxes, thereby providing a stricter geometric penalty. In terms of optimization efficiency, MPDIoU avoids the complex aspect ratio calculation used in CIoU by directly penalizing corner distances, thereby reducing computational complexity. This mechanism accelerates convergence during training and improves bounding box regression accuracy for animal motifs with diverse morphologies and complex contours. Therefore, this study replaces CIoU with MPDIoU as the loss function. Its calculation formulas are as follows:

(6) $$L_{MPDIoU}=1-IoU+\frac{d_{1}^{2}}{h^{2}+w^{2}}+\frac{d_{2}^{2}}{h^{2}+w^{2}}$$

(7) $$d_{1}^{2}={\left(x_{1}^{pred}-x_{1}^{gt}\right)}^{2}+{\left(y_{1}^{pred}-y_{1}^{gt}\right)}^{2}$$

(8) $$d_{2}^{2}={\left(x_{2}^{pred}-x_{2}^{gt}\right)}^{2}+{\left(y_{2}^{pred}-y_{2}^{gt}\right)}^{2}$$

where $d_{1}^{2}$ and $d_{2}^{2}$ denote the squared Euclidean distances between the top-left corners and the bottom-right corners of the predicted and ground truth boxes, respectively; h and w represent the height and width of the input image, respectively; $x_{1}^{pred},y_{1}^{pred}$) and ($x_{2}^{pred},y_{2}^{pred}$) indicate the coordinates of the top-left and bottom-right corners of the predicted box, respectively; and ($x_{1}^{gt}$,$y_{1}^{gt}$) and ($x_{2}^{gt}$,$y_{2}^{gt}$) indicate the coordinates of the top-left and bottom-right corners of the ground truth box, respectively.

### 2.3. Model Evaluation Metrics

To evaluate the improvement effect of the proposed model, this study adopts Precision, Recall, F1 score, and mean Average Precision (mAP) as the core evaluation metrics for the model’s performance.

Precision (P) refers to the model’s capability to correctly identify specific motif types and is defined as the proportion of correctly identified positive samples among all targets predicted as positive by the model. A higher precision indicates that the model has a lower false positive rate when detecting the animal motifs. Its calculation formula is as follows:

(9) $$P=\frac{TP}{TP+FP}$$

Recall (R) refers to the model’s capability to comprehensively detect specific motif types, i.e., the proportion of correctly identified positive samples out of all actual positive samples. A higher recall indicates that the model has a lower missed detection rate when detecting the animal motifs. Its calculation formula is as follows:

(10) $$R=\frac{TP}{TP+FN}$$

The F1 score is the harmonic mean of precision and recall, serving as one of the critical metrics for evaluating the comprehensive performance of the model. A higher F1 score indicates that the model achieves a better balance between reducing false positives and avoiding missed detections. Its calculation formula is as follows:

(11) $$F_{1}=\frac{2PR}{P+R}$$

Average Precision (AP) refers to the model’s detection performance for a single category of animal motifs, whilst mean Average Precision (mAP) represents the average of the APs across the five specific motif categories, comprehensively reflecting the model’s overall detection performance for multi-class motifs. This study employs the mAP metric under different IoU thresholds. Specifically, mAP@0.5 represents the average of the APs for all categories when the IoU threshold is set to 0.5, reflecting the model’s basic localization capability for the animal motifs. Furthermore, mAP@0.5:0.95 refers to the average of the mAPs calculated at various IoU thresholds ranging from 0.5 to 0.95 with a step size of 0.05. This metric reflects the model’s detection performance under different localization precision requirements and highlights its boundary-level regression capability for intricate motif contours. Its specific calculation formulas are as follows:

(12) $$\mathrm{AP}=\int_{0}^{1}P\left(R\right)\;dR$$

(13) $$mAP=\frac{1}{N}\sum_{i=1}^{N}{AP}_{i}$$

where TP is the number of positive samples correctly identified as positive by the model; FP is the number of negative samples incorrectly identified as positive by the model; FN is the number of positive samples incorrectly identified as negative by the model; N represents the total number of detection categories; $P\left(R\right)$ represents Precision as a function of Recall.

### 2.4. Experimental Environment and Parameter Settings

The experiments in this study were implemented using the Ultralytics package, version 8.0.141, with the YOLOv8n model architecture. The experimental environment used the Windows 10 64-bit operating system, and the hardware platform was equipped with an NVIDIA RTX 4060 Ti GPU (NVIDIA Corporation, Santa Clara, CA, USA) with 8 GB of video memory. The software environment was configured with Python 3.8, PyTorch 1.10, and CUDA 11.3. During model training, the input image size was uniformly resized to 640 × 640 pixels, the number of training epochs was set to 200, and the batch size was set to 4. The optimizer was SGD, with the initial learning rate (lr0) set to 0.01, the final learning-rate factor (lrf) set to 0.01, and the weight decay set to 0.0005.

The batch size of 4 was determined after multiple rounds of parameter tuning under the available hardware conditions. When training on an 8 GB GPU, a larger batch size substantially increased GPU memory consumption, especially after the introduction of the DCNv2 and EMA modules. Although a smaller batch size may increase the noise in gradient estimation and make the BatchNorm statistics relatively less stable, the baseline model and all ablation-study models in this study were trained using the same batch-size setting to ensure consistency in the training conditions across different experiments.

To reduce the influence of random initialization, data-loading order, and stochastic perturbations during the training process on the experimental results, five independent repeated training runs were conducted for both the original YOLOv8n model and the improved model under the same dataset split, training parameters, data augmentation strategy, and pretrained-weight settings. The random seeds were set to 0, 1, 2, 3, and 4. In each training run, the best-performing model weights were selected based on the performance on the validation set, and the corresponding performance was then recorded on the same fixed validation set. The repeated-run results are reported as the mean and standard deviation of the five repeated experiments.

## 3. Results

### 3.1. Quantitative Evaluation of Detection Performance

**Figure 7:**
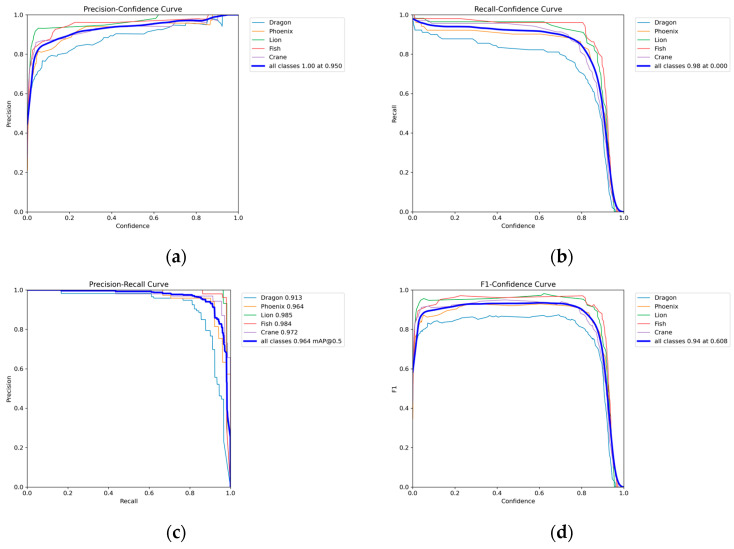
Performance evaluation curves of the improved YOLOv8n model: (**a**) Precision-Confidence (P-Confidence) curve; (**b**) Recall-Confidence (R-Confidence) curve; (**c**) Precision-Recall (P-R) curve; (**d**) F1-Confidence curve.

By integrating the Precision-Confidence (P-Confidence), Recall-Confidence (R-Confidence), Precision-Recall (P-R), and F1 score curves, this study comprehensively evaluates the detection capability of the improved YOLOv8n model. As indicated by the P-Confidence curve (Figure 7a), the curves for all categories exhibit a rapid upward trend in the lower confidence intervals and maintain a high and stable level across a broad range of thresholds. When the confidence threshold is set to 0.950, the mean precision across all categories reaches 1.00. As shown by the R-Confidence curve (Figure 7b), under the condition where the confidence threshold approaches 0, the mean recall across all categories achieves 0.98. As the threshold gradually increases, the recall exhibits a smooth decay trend. Among them, the decay for the lion, fish, and phoenix motifs is relatively gentle, whereas the overall recall performance for the dragon motif is relatively weak. The P-R curve (Figure 7c) further quantifies the model’s overall detection capability across different categories. The mean Average Precision (mAP@0.5) across all categories reaches 0.964, indicating strong overall detection performance. Specifically, the lion and fish motifs yield the highest AP values, reflecting the network’s stable feature representation capability for these categories; in contrast, the AP value for the dragon motif is the lowest, at 0.913 (Figure 7c). As illustrated by the F1 score curve (Figure 7d), which serves as the harmonic mean of precision and recall, when the confidence threshold is set to 0.608, the mean F1 score across all categories reaches 0.94. This result indicates that the model achieves a balanced trade-off between precision and recall under the tested conditions. Overall, these multi-dimensional metrics show that the improved model can effectively extract and recognize the visual features of animal motifs.

### 3.2. Model Classification Accuracy Analysis

**Figure 8:**
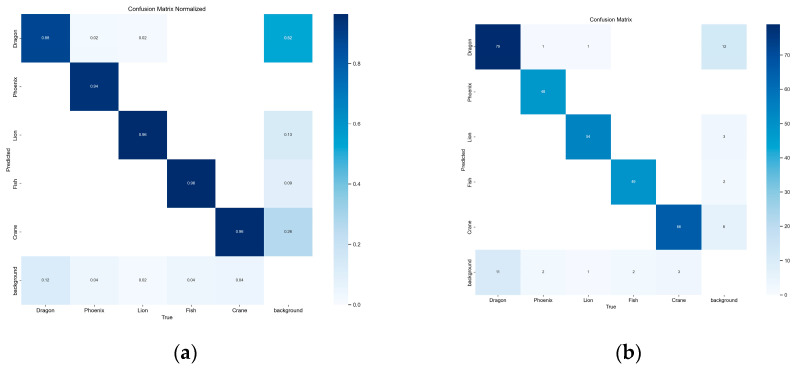
Confusion matrices of the improved YOLOv8n model: (**a**) Normalized confusion matrix; (**b**) Unnormalized confusion matrix.

To evaluate the specific performance and error distribution of the improved YOLOv8n model on various categories of the animal motifs, this study generated the normalized confusion matrix (Figure 8a) and the unnormalized confusion matrix (Figure 8b). As indicated by the diagonal values in Figure 8a, the model demonstrates strong classification and detection performance across the five animal motif categories, with recall maintained at a relatively high level. Specifically, the model achieves the best recognition performance for the lion, fish, and crane motifs, each with a recall of 0.96. The phoenix motif follows with a recall of 0.94, while the dragon motif achieves a recall of 0.88. The unnormalized confusion matrix (Figure 8b) further corroborates this result, demonstrating that the model correctly identified 79 dragon, 48 phoenix, 54 lion, 49 fish, and 66 crane instances. Furthermore, the matrices reveal minimal inter-class misclassifications, indicating that the model possesses outstanding class discrimination capabilities and can effectively overcome the interference caused by inter-class morphological similarities.

### 3.3. Model Convergence Analysis

**Figure 9:**
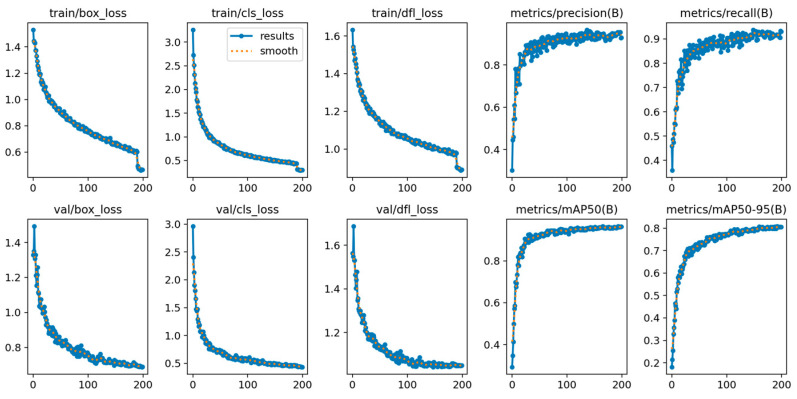
Training and validation loss curves alongside performance evaluation metrics of the improved YOLOv8n model.

This study recorded the dynamic evolution process of the loss functions and evaluation metrics of the improved YOLOv8n model over 200 training epochs (Figure 9). Observing the three left-hand columns of the chart, the bounding box regression loss (box_loss), classification loss (cls_loss), and distribution focal loss (dfl_loss) for both the training and validation sets exhibited an extremely steep downward trend during the initial 50 epochs. As training progressed, the loss curves gradually became stable, indicating that the model effectively learned the core visual features of the animal motifs. Throughout the 200-epoch training process, the validation loss converged consistently with the training loss, with no obvious divergence or rebound. This suggests that the model maintained stable performance during training, with no evident overfitting. In the metric curves, Precision and Recall increased rapidly during the first 50 epochs and then gradually stabilized at a high level. The mAP@0.5 converged to above 0.96, while mAP@0.5:0.95 stabilized at approximately 0.80. These results indicate that the improved model achieved stable convergence and demonstrated effective classification and localization performance for animal motif detection.

### 3.4. Results of the Ablation Study

To verify the effectiveness of the proposed improved YOLOv8n model, this study designed five sets of ablation experiments to independently evaluate the performance of the baseline YOLOv8n and its variations following the integration of different module combinations. As indicated by the data in Table 3, the introduction of each individual module generated a distinct positive gain for the network’s performance. Notably, the integration of Deformable Convolution Networks v2 (DCNv2) yielded the most significant performance enhancement. Finally, when all three improvement mechanisms were simultaneously integrated into the network, the model achieved optimal performance, exhibiting a pronounced synergistic effect. Specifically, the Precision, Recall, mAP@0.5, and mAP@0.5:0.95 of the optimized model reached 94.8%, 90.7%, 96.4%, and 80.8% respectively. These metrics represent significant increases of 2.5%, 2.5%, 1.7%, and 3.2% over the baseline model, thereby comprehensively validating the enhancement efficacy of the combined modules. Furthermore, although the inference speed of the final integrated model experienced a slight decline due to increased computational overhead, the frame rate of the improved model remained stable at 89.3 FPS. This comfortably satisfies the conventional requirements for real-time detection, successfully achieving a reasonable balance between high detection accuracy and high inference efficiency.

### 3.5. Stability Analysis Across Repeated Runs

Table 4 reports the mean values and standard deviations of the performance of the two models on the validation set over five independent experimental runs. The original YOLOv8n model achieved mAP@0.5 and mAP@0.5:0.95 values of 94.7% ± 0.2% and 77.6% ± 0.2%, respectively, whereas the improved model achieved corresponding values of 96.4% ± 0.2% and 80.8% ± 0.2%. The results show that the improved model consistently obtained higher average detection performance under different random initialization conditions, indicating that the observed performance improvement was not caused by incidental fluctuations from a single training run.

### 3.6. Visualisation Analysis of Recognition Results

**Figure 10:**
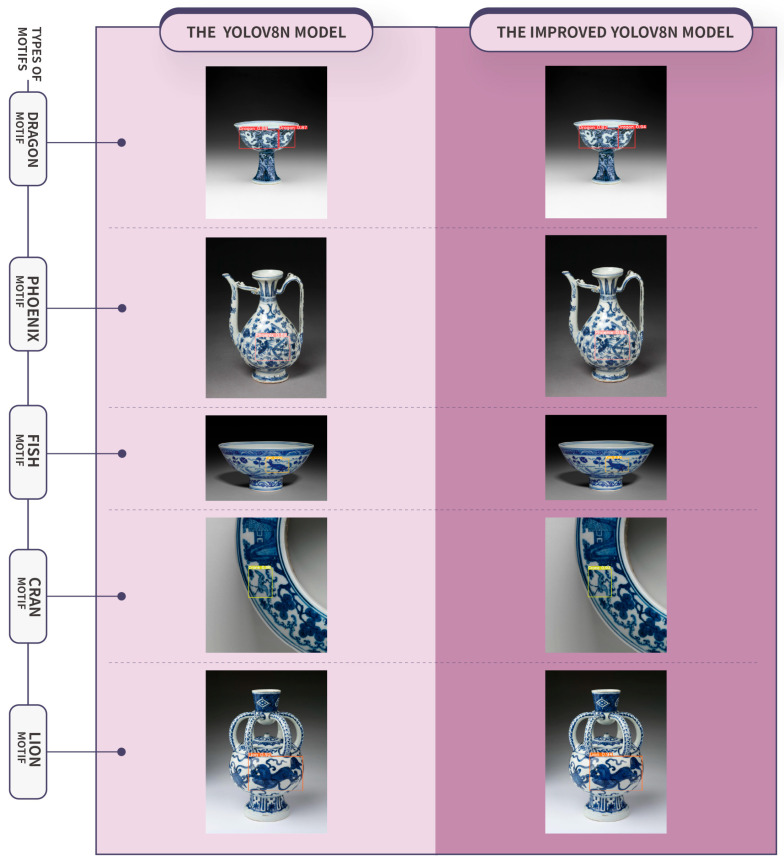
Visual comparison of the detection results between the baseline YOLOv8n model and the improved model across the five categories of animal motifs.

To further evaluate the detection performance of the proposed model, this section uses the baseline YOLOv8n model for comparative visualization. As shown in Figure 10, the visualization results present the bounding box localization results and corresponding confidence scores generated by the baseline and improved models for the five animal motif categories on intact vessels. Compared with the baseline YOLOv8n model, the improved model achieves higher confidence scores and more stable localization performance across the five motif categories. These results indicate that the three introduced mechanisms help improve detection performance under complex visual conditions, including curved-surface deformation, blurred underglaze boundaries, and interference from monochrome decorative backgrounds.

**Figure 11:**
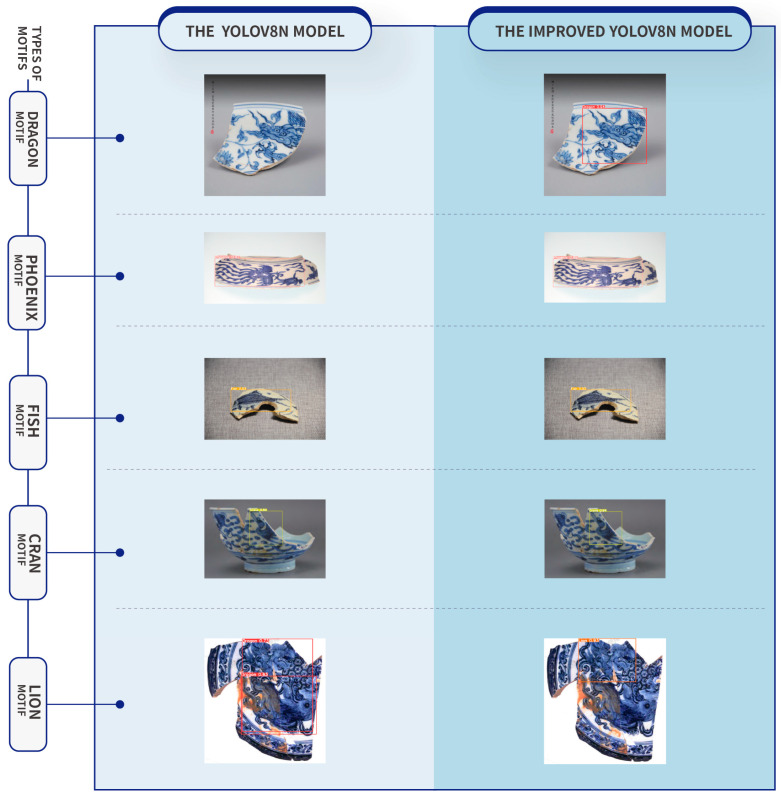
Visual comparison of recognition performance between the baseline YOLOv8n model and the improved model on incomplete blue and white porcelain shards.

To further examine the robustness of the improved model under non-ideal image conditions, this study conducted additional visual tests on Blue and White Porcelain shard samples with incomplete morphologies and substantial information loss. Figure 11 compares the detection results of the baseline YOLOv8n model and the improved model on shard images. The results show that the baseline YOLOv8n model exhibited missed detections and misclassifications in some shard samples. For example, in the dragon motif shard samples, the baseline model missed certain targets and incorrectly identified some lion motif shards as dragon motifs. In contrast, the improved model showed better local feature discrimination and more stable recognition performance. These findings suggest that the improved model can better capture surviving local motif features in damaged porcelain shard images, supporting its potential use in image-based recognition tasks involving incomplete ceramic samples.

### 3.7. External Test Set Evaluation for Generalization

To further evaluate generalization performance, an independent external test set containing 168 Blue and White Porcelain images with complete bounding-box annotations was constructed. These images were not included in the original dataset and were not used for training, validation, internal testing, model selection, or hyperparameter tuning. The best model weights selected using the internal validation set were directly evaluated on this external test set without additional fine-tuning.

As shown in Table 5, the improved model achieved Precision, Recall, mAP@0.5, and mAP@0.5:0.95 values of 91.4%, 87.3%, 93.2%, and 76.1%, respectively, on the independent external test set, all of which were higher than those of the original YOLOv8n model. Although the performance on the external test set was lower than that on the internal validation set, the improved model still maintained favorable detection performance on unseen external images. Together with the internal validation results, these findings indicate that the introduced DCNv2, EMA, and MPDIoU modules not only improve the model’s recognition performance on internal validation data, but also help it retain a certain level of recognition and localization capability on unseen Blue and White Porcelain animal motif images.

### 3.8. Edge-Device Deployment Evaluation

OpenVINO, version 2024.1.0 is a cross-platform deep learning toolkit developed by Intel, mainly used to optimize the inference performance of neural networks on hardware platforms such as CPUs and integrated GPUs. To further evaluate the practical deployment feasibility of the improved model on edge-computing devices, this study deployed the model on an Intel NUC10i7FNH compact computing platform. This platform is equipped with an Intel Core i7-10710U processor (Intel Corporation, Santa Clara, CA, USA) and Intel UHD Graphics integrated GPU (Intel Corporation, Santa Clara, CA, USA), with physical dimensions of 117 mm × 112 mm × 51 mm, providing a relatively compact hardware environment for image inference in edge-computing scenarios.

**Figure 12:**
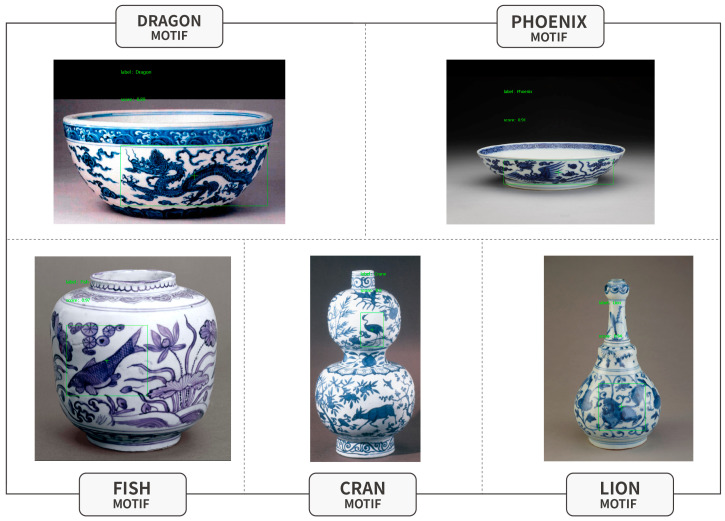
Representative edge-device inference results of the improved YOLOv8n model.

On this hardware platform, the edge-device inference test results for Blue and White Porcelain animal motif images are shown in Figure 12. The improved model has a computational cost of 7.1 GFLOPs, and the exported model file size is 11.7 MB. When processing a single motif image, the inference time of the model on the CPU and integrated GPU was 115 ms and 75 ms, respectively. These results indicate that the improved model can perform image-based detection of animal motifs on Blue and White Porcelain images on a compact edge-computing platform, with acceptable inference efficiency.

## 4. Discussion

### 4.1. Foundation Established by the Dedicated Dataset and Targeted Data Augmentation Strategies

High-quality annotated datasets are not only the foundation for evaluating algorithm performance but also drive object detection algorithms towards increasingly complex and challenging directions [51]. Currently, mainstream publicly available datasets for general object detection primarily include the VOC and COCO datasets [52,53]. Although these datasets contain large numbers of images from everyday scenes, they show a clear domain gap when applied to the image-based recognition of intricate animal motifs on specific ceramic objects. Therefore, they cannot provide sufficient domain-specific data support for ceramic image analysis [37,54]. To address this limitation, this study constructed a dedicated annotated image dataset of animal motifs on Ming Dynasty Blue and White Porcelain. To improve the representativeness of the dataset, the collected samples cover different vessel forms, imaging perspectives, preservation conditions, and visual scales. These factors are closely related to the main challenges of motif recognition in ceramic images, including geometric deformation, illumination changes, scale variation, and background interference. In addition, many Blue and White Porcelain objects have been affected by physical wear, fragmentation, and surface degradation during long-term circulation and preservation. Therefore, the dataset was not limited to intact vessels with clear motifs. It also included samples with blurred motifs and shard images with incomplete decorative information. This design allows the dataset to better reflect non-ideal image conditions encountered in practical ceramic image analysis.

On this basis, this study applied targeted data augmentation strategies to the training set, including random cropping, random rotation, brightness adjustment, and Gaussian noise. These operations were used to increase training-sample diversity under controlled visual perturbations and to improve the model’s robustness to scale variation, viewpoint changes, illumination differences, and image noise. The experimental results suggest that, for animal motif images with unstable visual features and varied preservation conditions, targeted data augmentation can improve the reliability of object detection performance. Overall, the dedicated annotated image dataset constructed in this study addresses the scarcity of specialized image datasets for animal motif recognition on Ming Dynasty Blue and White Porcelain and provides a data basis for subsequent research on image-based motif recognition and ceramic image analysis.

### 4.2. Effectiveness of the Three Improvement Mechanisms in Enhancing Detection Accuracy

To address the three major challenges of geometric distortion, background confusion, and edge blurring in the recognition of the animal motifs, this study innovatively integrates three core improvement modules—DCNv2, EMA, and MPDIoU—into the baseline model. These three mechanisms not only exert their unique advantages independently but also form a robust complementarity during the feature extraction and bounding box regression processes, synergistically elevating the overall detection performance of the model.

Firstly, the CBS modules in the Backbone and Neck networks are replaced with Deformable Convolution Networks v2 (DCNv2). Traditional convolutions possess inherent limitations when handling geometric transformations. In contrast, the animal motifs are typically small targets with complex curved contours, distributed across the curved surfaces of diverse vessel shapes (e.g., bowls, plates, and vases). Consequently, they are highly susceptible to pronounced morphological stretching and geometric distortion under varying shooting angles. Following the introduction of DCNv2, the network’s adaptability to irregular targets is significantly enhanced, particularly strengthening the feature extraction capability for small targets, thereby effectively improving the model’s detection performance [55,56,57]. Experimental results demonstrate that this modification substantially augments the model’s geometric perception of irregular contours and deformed motifs, effectively mitigating feature extraction biases induced by curved surface projections.

Secondly, the Efficient Multi-scale Attention (EMA) mechanism is integrated into the Neck network. Foreground–background entanglement has also been identified as a common challenge in object detection under complex visual conditions, such as small or partially occluded target detection in cluttered natural backgrounds [58]. In the present study, animal motifs are frequently intertwined with intricate auxiliary decorative patterns, resulting in visual confusion between the foreground motifs and background ornamentation. Through multi-scale receptive fields constructed by parallel sub-networks, the EMA mechanism captures spatial information at varying granularities and establishes interdependencies across different spatial locations. This facilitates cross-spatial learning, effectively suppressing interference from complex background patterns and lowering the missed detection rate under complex background conditions [59,60].

Finally, the CIoU loss function is replaced with the MPDIoU loss function. During the firing process of blue and white porcelain, the underglaze cobalt pigment frequently diffuses, resulting in blurred motif boundaries and making the true contours challenging to accurately define. Under such conditions, CIoU is prone to regression instability when handling targets with indistinct edges or substantial scale variances. The MPDIoU function introduces the Euclidean distance between the top-left and bottom-right corners of the predicted and ground truth boxes as an additional geometric constraint, thereby improving the alignment accuracy and convergence stability of the bounding boxes.

Synthesizing the aforementioned three improvement strategies, the model overcomes the limitations of any single mechanism and yields a pronounced synergistic effect. As evidenced by the experimental data, the improvement mechanisms bring about a comprehensive leap in detection performance. Most notably, the stringent mAP@0.5:0.95 metric demonstrates a substantial increase of 3.2% compared to the baseline model. Simultaneously, whilst maintaining high precision, the model sustains an inference speed of 89.3 FPS, achieving a reasonable balance between the accuracy of image-based recognition of intricate animal motifs and the inference efficiency required for practical applications.

### 4.3. Robustness of the Improved Model Under Incomplete and Non-Ideal Image Conditions

Experimental results demonstrate that the proposed model maintains strong robustness even when motifs are blurred or partially missing. The model can effectively extract surviving local textural information by capturing fine-grained features, such as dragon scales, phoenix tails, and lion fur, rather than relying solely on complete morphological structures. This capability holds significant implications for practical archaeological and museum scenarios. In actual archaeological environments, blue and white porcelain frequently exists in the form of shards, with motifs often exhibiting wear and fragmentation; intact and clear samples are relatively scarce. Consequently, the aforementioned results indicate that the proposed model possesses substantial potential for real-world application scenarios, including the recognition of archaeological shards, digital archiving, and the facilitation of cultural artefact restoration.

### 4.4. Limitations of the Improved Model in the Recognition of Complex Dragon Motifs

Although the improved model achieves strong overall detection performance, the recognition of dragon motifs remains more challenging than that of the other animal motif categories. The experimental results show that the dragon motif category has the lowest AP value of 0.913 and a recall value of 0.88, indicating that there is still room for improvement in detecting motifs with highly complex morphologies. This weaker performance is closely related to the inherent visual characteristics of dragon motifs on Ming Dynasty Blue and White Porcelain. First, dragon motifs show substantial intra-class variation. They are not represented by a single stable visual form, but include multiple compositional subtypes, such as yunlong (雲龍), kuilong (夔龍), yinglong (應龍), and naochao long (鬧潮龍). These subtypes differ considerably in body posture, head structure, claw form, surrounding decorative elements, and spatial arrangement. As a result, samples belonging to the same dragon category may still present highly heterogeneous visual features, which increases the difficulty of learning stable category-level feature representations. Second, compared with other animal motifs, dragon motifs are often densely intertwined with complex auxiliary decorative patterns, such as auspicious clouds and seawater-wave patterns. This foreground–background entanglement makes it difficult for the model to distinguish the dragon body, claws, and surrounding decorative elements, thereby reducing localization confidence and increasing the risk of missed detections. Future work will therefore collect more dragon motif samples from different compositional subtypes and painting techniques, and will consider subtype-level annotation, targeted augmentation of hard samples, and finer-grained feature learning strategies to improve the detection of highly heterogeneous dragon motifs.

## 5. Conclusions

This study proposes an image-based recognition method for intricate animal motifs on Ming Dynasty Blue and White Porcelain using an improved YOLOv8n-based object detection framework. By constructing a dedicated annotated image dataset of animal motifs, applying targeted data augmentation strategies, and integrating DCNv2, EMA, and MPDIoU into the baseline model, the proposed method addresses several challenges in ceramic image recognition, including geometric deformation, background interference, scale variation, and boundary ambiguity. The proposed method provides a non-destructive and real-time approach for the image-based recognition of intricate decorative motifs in ceramic images. It may support future work in ceramic image analysis, digital documentation, museum collection management, and image-based visual sensing applications.

Nevertheless, the dataset used in this study remains relatively limited in scale compared with large-scale benchmark datasets commonly used in general object detection, primarily due to the availability and preservation conditions of surviving Ming Dynasty Blue and White Porcelain objects. Future research will focus on further expanding the dataset by increasing the number of samples, incorporating more diverse preservation states and vessel forms, and adding additional motif categories such as plant motifs and narrative figure motifs. Image data from other historical periods will also be included to improve the recognition scope and cross-period generalization capability of the model.

## Figures and Tables

**Table 1:** Categories, symbolic meanings, and distribution of original image samples across the training, validation, and test sets.

Category	Sample Image	Symbolic Meaning	Number of Original Images	Training Images	Validation Images	Test Images
Dragon motif	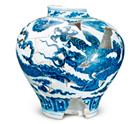	Authority and order	335	234	67	34
Phoenix motif	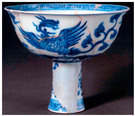	Auspiciousness and tranquility	146	102	29	15
Fish motif	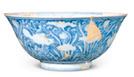	Abundance and prosperity	128	89	26	13
Crane motif	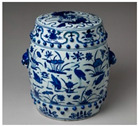	Longevity and purity	116	81	23	12
Lion motif	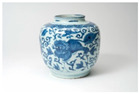	Majesty and protection	106	74	21	11
Total			831	580	166	85

**Table 2:** Visual examples of the original images and their corresponding results under different data augmentation methods.

Category	Original Image	Random Cropping	Random Rotation	Brighten	Darken	Gaussian Noise
Dragon Motif	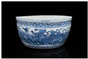	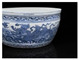	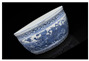	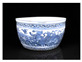	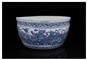	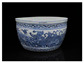
Phoenix Motif	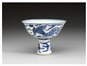	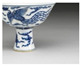	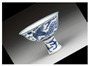	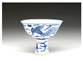	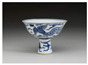	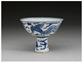
Fish Motif	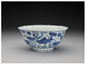	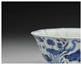	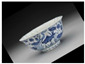	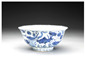	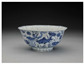	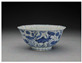
Crane Motif	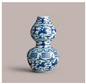	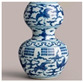	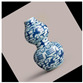	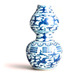	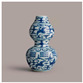	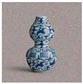
Lion Motif	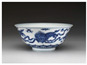	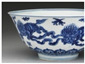	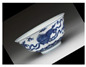	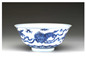	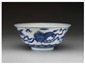	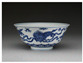

**Table 3:** Ablation study results of different module combinations in the improved YOLOv8n model.

Modules	Precision	Recall	mAP@ 0.5	mAP@ 0.5:0.95	FPS	GFLOPs
YOLOv8n	92.3%	88.2%	94.7%	77.6%	91.4	8.9
YOLOv8n-DCNv2	93.9%	89.7%	95.6%	79.7%	89.6	7.8
YOLOv8n-EMA	93.5%	89.4%	95.3%	79.4%	89.7	8.2
YOLOv8n-MPDIoU	92.8%	88.6%	94.9%	78.3%	90.2	8.9
YOLOv8n-DCNv2-EMA-MPDIoU	94.8%	90.7%	96.4%	80.8%	89.3	7.1

**Table 4:** Detection performance stability of the original YOLOv8n and improved YOLOv8n across five independent runs with different random seeds.

Model	Metric	Seed 0	Seed 1	Seed 2	Seed 3	Seed 4	Mean ± SD
YOLOv8n	mAP@0.5 (%)	94.6	94.9	94.5	94.8	94.7	94.7 ± 0.2
	mAP@0.5:0.95 (%)	77.6	77.9	77.3	77.7	77.5	77.6 ± 0.2
Improved YOLOv8n	mAP@0.5 (%)	96.3	96.5	96.1	96.6	96.4	96.4 ± 0.2
	mAP@0.5:0.95 (%)	80.6	81.1	80.5	80.9	80.8	80.8 ± 0.2

**Table 5:** Detection performance comparison between the original YOLOv8n model and the improved model on the independent external test set.

Model	Precision (%)	Recall (%)	mAP@0.5 (%)	mAP@0.5:0.95 (%)
YOLOv8n	88.6	84.7	90.9	72.8
Improved YOLOv8n	91.4	87.3	93.2	76.1

## Data Availability

The data presented in this study are available on request from the corresponding author. The data are not publicly available due to ongoing research.
